# Case Report: Assessment of primary myxofibrosarcoma in the left atrium using multimodal ultrasonography

**DOI:** 10.3389/fcvm.2024.1378655

**Published:** 2024-05-17

**Authors:** Liyan Hu, Zhaohui Wang, Yu Chen, Xiaowei Zhang

**Affiliations:** ^1^ Department of Ultrasonography, People’s Hospital of Dong yang, Dong yang, Zhejiang, China; ^2^ Department of Cardiac Surgery, People’s Hospital of Dong yang, Dong yang, Zhejiang, China; ^3^ Department of Pathology, People’s Hospital of Dong yang, Dong yang, Zhejiang, China

**Keywords:** myxofibrosarcomas, cardiac tumor, multimodal ultrasonography, contrast-enhanced ultrasound (CEUS) imaging, cardiac surgery

## Abstract

Primary myxofibrosarcoma of the heart, a rare cardiac malignancy, was diagnosed in a middle-aged female patient exhibiting progressive dyspnea following transthoracic echocardiography and pathological analysis. Postoperatively, the patient underwent chemotherapy and Lenvatinib mesylate therapy, with regular check-ups confirming her survival. After 10 months the patient is still alive and well.

## Introduction

Primary cardiac tumors are extremely rare, with a reported prevalence of 0.002% to 0.03% in postmortem studies. Myxomas account for approximately 25% of all primary cardiac tumors, while the remainder are benign (1). The majority (95%) of these primary malignant tumors are vascular sarcomas (2). Fibrosarcoma, also known as myxofibrosarcoma, is a rare tumor that can manifest in any chamber of the heart but is most commonly found in the left atrium. The tumor tissue is characterized by the presence of a mucinous stroma (3). Primary cardiac myxofibrosarcoma is very aggressive. Although only a few cases have been reported, studies have shed light on clinical presentation, pathological features, and treatment strategies (4–7). Moreover, the diagnostic value of ultrasonography, particularly cardiac contrast echocardiography, has not been widely discussed.

## Case presentation

A 59-year-old woman presented with a two-month history of chest tightness and shortness of breath that worsened significantly in the last 12 h prior to admission. Previous episodes of chest discomfort and dyspnea occurred during physical activity and were alleviated by rest. Upon admission, she was experiencing orthopnea and acute emesis. The patient had a past history of hypertension and diabetes for 2 years. Her breathing sounded slightly rough. Her heart rate was at 104 beats per min, and she had a rhythmic systolic rumble (grade 2/6) in the mitral region but no lower limb swelling.

Laboratory results indicated a serum troponin level of 0.043 ng/ml, normal serum creatinine, and an elevated B-type natriuretic peptide (BNP) level of 3,170 pg/ml. A transthoracic echocardiogram revealed two masses in the left atrial cavity. The larger mass measured 7.2 × 3.2 cm and was located proximally to the mitral valve. The smaller lesion measured 2.5 × 2.4 cm and was directly linked to the mitral valve. The masses were characterized by irregular shapes, clear boundaries, and uneven echogenicity. During diastole, the small mass prolapsed into the left ventricular inflow tract, generating a 35 mmHg pressure differential. The transthoracic echocardiogram also found mitral orifice obstruction with mild regurgitation; moderate pulmonary hypertension; mild tricuspid regurgitation; small amount of pericardial effusion, Pressure difference halved time 149 ms, and no significant abnormalities were observed in the residual valves. Some specific data is shown in Table 1. Based on ultrasound findings, a preliminary diagnosis of a left atrial mass was made, with suspicion of malignancy.

**Table 1:** Transthoracic echocardiogram data.

Name	Value	Name	Valve
Ejection fraction	74%	Left atrium	41 mm
Fraction shortening	41%	Right atrium	40 mm
Left ventricular end-diastolic volume	32 mm	Pulmonary artery systolic pressure	74 mmHg
Mitral valve orifice area	1.48 cm^2^	Mean mitral valve pressure gradient	35 mmHg
Maximum diastolic mitral orifice flow rate	4.02 m/s	Maximum velocity of tricuspid regurgitation	4.23 m/s

**Figure 1:**
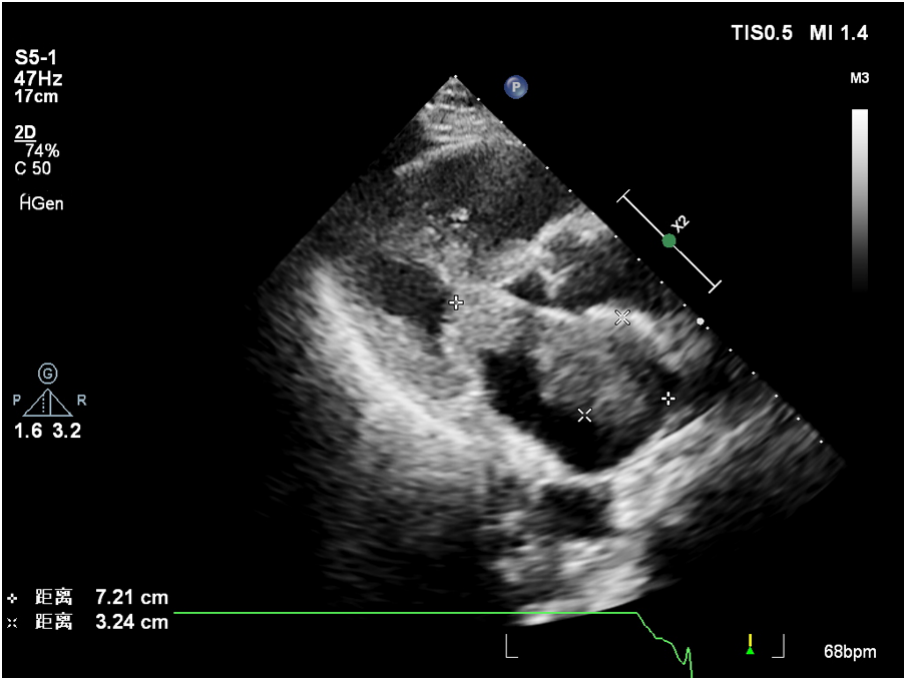
The larger mass close to the mitral valve.

Contrast-enhanced echocardiography was recommended, revealing rapid perfusion of the contrast material into the right and left atria and ventricles. After a few cardiac cycles, the mass was substantially enhanced, almost matching the surrounding myocardium, confirming the likelihood of a malignant tumor (Figure 1, Supplementary Video 1).

**Figure 2:**
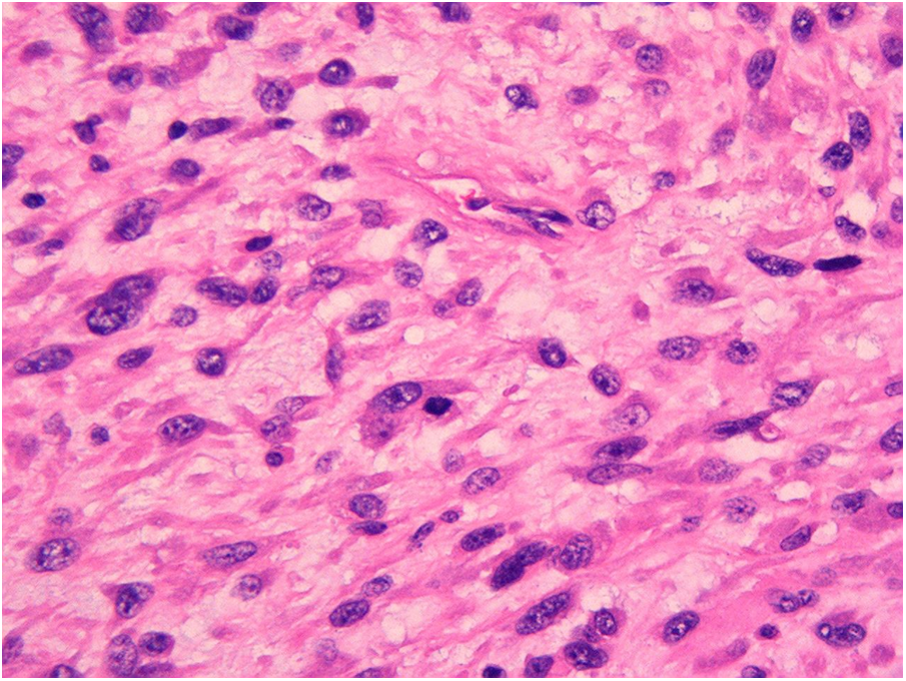
Primary myxofibrosarcoma of the left atrium.

**Figure 3:**
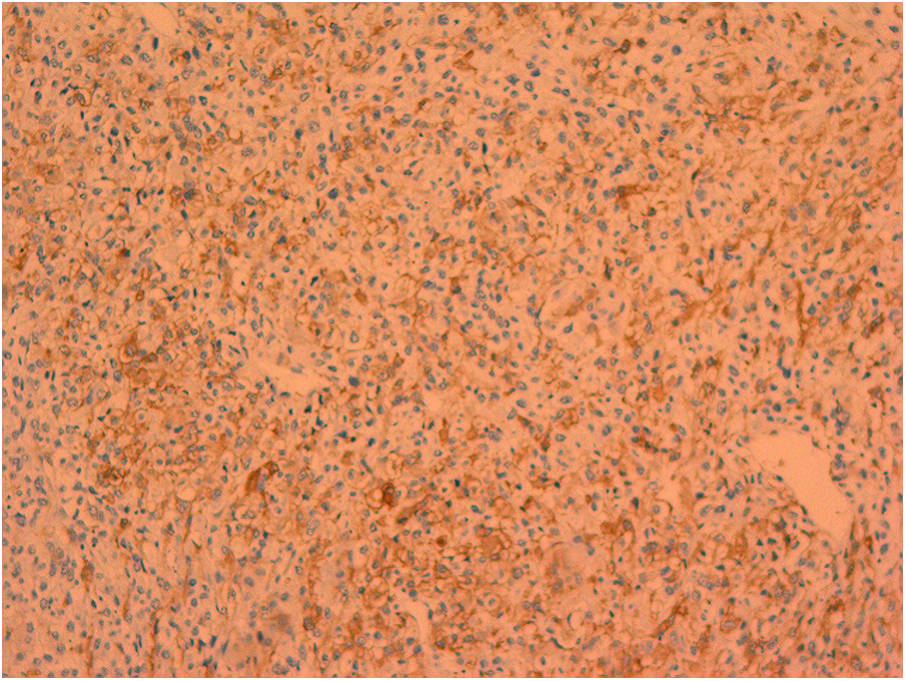
Smooth muscle actin positive.

On February 8, 2023, the patient underwent surgery for the left atrial lesions. The surgical approach was median sternotomy. cardiopulmonary bypass was used in the surgery through Aortic Cannulation and bicaval cannulation. More than 2 lesions, with different sizes, were found in the left atrial. The cardiac tumors exhibited fragility and spanned from the roof of the left atrium to the edge of the mitral valve. The left atrial tumors were excised en block, accompanied by the removal of a portion of the endocardium. Finally, a 3 cm × 4 cm size Cow pericardial patch was used to rebuild the atrial septum. Subsequent to the surgical procedure, postoperative pathology analysis identified a cardiac malignant tumor consistent with myxofibrosarcoma. This diagnosis was substantiated through immunohistochemistry and microscopic morphology examination (Figures 2, 3). Immunohistochemical staining results were as follows: CK(AE1/AE3)(−), EMA(−), Vimentin(+), CD31(−), CD34(−), D2-40(−), Desmin(−), SMA(small amount+), MyoD1(−), S-100(−), Ki-67(+ approximately 30%); F8(−), Actin(−), MSA(−), caldesmon(+), B-catenin(−), HMB45(−), SOX-10(−), CD117(−), DOG-1(−), TFE3(+).calretinin(−), WT-1(+), Bcl-2(−), CD99(+).

The patient recovered well after surgery without any surgical complications. The symptoms of chest tightness and shortness of breath disappeared when the patient was discharged. PET-CT examination was performed after the operation, revealing that the patient had multiple metastatic lesions in the liver and left femur. Subsequently, the patient underwent 6 cycles of chemotherapy, comprising albumin-bound Paclitaxel 200 mg, Liposomal Doxorubicin 40 mg, and Anlotinib 10 mg.

**Figure 4:**
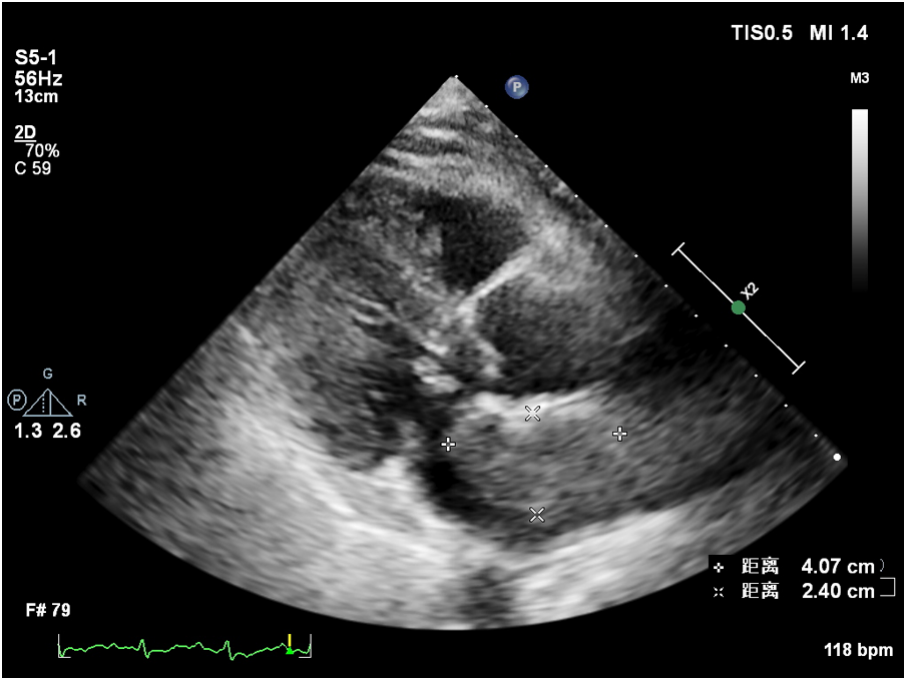
Recurrent tumor in the left atrium.

At 8 months post-surgery, a 41 × 24 mm mass in the left atrium was detected using transthoracic echocardiography. The lesion had an irregular shape, uneven echogenicity, and an ill-defined boundary with the atrial septum. The maximum velocity of blood flow through the mitral valve was measured to be 1.39 meters per s. The maximum and mean pressure gradients across the valve were found to be 8 and 4 mmHg, respectively. During the contrast-enhanced ultrasound examination, perfusion of the contrast agent was observed in the mass. However, the enhancement degree was lower than that of the adjacent myocardium, leading to the diagnosis of tumor recurrence. (Figure 4, Supplementary Video 2). Considering tumor recurrence, Lenvatinib Mesylate was added as an anti-tumor therapy. The patient was followed up monthly in our hospital. Having gone through the series of treatments (Table 2), she complained of nothing but feeling a little weakness with firm support from family members and meticulous care from medical staff, she kept an Optimistic attitude.

**Table 2:** A timeline with relevant data.

Time	Event
2023-02-05	Admitted to the Cardiology Department
2023-02-06	An echocardiogram revealed two masses in the left atrial cavity
2023-02-07	Coronary angiography results in negative
2023-02-07	Transferred to the Cardiac Surgery Department
2023-02-08	Cardiac tumor resection surgery. Frozen sections indicated that mesenchymal tumor flap cells were abundant, necrotic, and tended to be borderline or low-grade malignant.
2023-02-17	Primary Myxofibrosarcoma was substantiated through immunohistochemistry and microscopic morphology examination; Discharged.
2023-03-21	PET-CT revealed that the patient had multiple metastatic lesions in the liver and left femur.
From 2023 to 04 to 2023-10	6 cycles of chemotherapy: Paclitaxel 200 mg, Liposomal Doxorubicin 40 mg, and Anlotinib 10 mg.
2023-10	Routine outpatient follow-up. An echocardiogram revealed a recurrent mass in the left atrial cavity. Lenvatinib Mesylate was added as an anti-tumor therapy.
From 2023 to 11 to 2024-03	Routine outpatient follow-up.

## Discussion

Left atrial myxofibrosarcoma is usually characterized by low or moderate echogenic masses attached to the left atrial valve or wall (3). These lesions often exhibit unclear boundaries from surrounding normal tissue, irregular shapes, and uneven echogenicity. Additionally, they are frequently large, sessile, and possess a broad base. They may also invade the pulmonary veins and exhibit other features. In the present case, one of the masses oscillated around the mitral valve orifice during the cardiac cycle, obstructing the valve orifice during diastole. This resulted in symptoms of mitral stenosis, such as chest tightness and dyspnea. Distinguishing left atrial myxofibrosarcoma from left atrial myxoma is crucial. The American Society of Echocardiography guidelines recommend the differentiation of intracardiac masses using ultrasound enhancement agents (8). Benign cardiac tumors, predominantly myxomas, typically exhibit sparse neovascularization, with the myxoid matrix composed of acidic mucopolysaccharides (9, 10). In contrast, malignant tumors, such as myxofibrosarcomas, grow rapidly and feature abundant new blood vessels, densely distributed with a dilated lumen, resulting in significant enhancement during angiography (11). Of note, conventional ultrasonography revealed that the mass had an irregular shape, uneven echogenicity, no peduncle, and a wide base, which required differentiation from a thrombus. Of note, thrombi do not exhibit enhanced imaging due to a lack of blood vessel supply, a feature distinguishing them from malignant tumors (8, 12). Postoperatively, a follow-up transthoracic echocardiography was conducted 8 months later, unveiling the recurrence of the tumor in the left atrium. Further characterization through contrast-enhanced ultrasound indicated sparse enhancement of the left atrial mass, with the degree of enhancement lower than that observed in the surrounding myocardium.

This observation contrasts with the preoperative contrast-enhanced ultrasonography, which demonstrated significant enhancement in the mass akin to the adjacent myocardium. The altered enhancement pattern may be attributed to the effects of chemotherapy and Lenvatinib Mesylate. Lenvatinib Mesylate is a tyrosine kinase (RTK) receptor inhibitor that inhibits the kinase activity of the vascular endothelial growth factor (VEGF) receptors VEGFR1(FLT1), VEGFR2(KDR) and VEGFR3(FLT4). Moreover, it also inhibits RTKs involved in other proangiogenic and tumorigenic pathways, including fibroblast growth factor (FGF), receptors FGFR1, 2, 3, and 4, and platelet-derived growth factor (PDGF) receptors PDGFRa, KIT, and RET.

According to case reports and guidelines, the most widely adopted treatment strategies are surgery combined with chemotherapy and targeted anti-tumor drugs (3, 7, 8, 10). However the outcomes are not satisfactory, the median survival for patients that had complete surgical resection was 17 months compared to 6 months with incomplete resection. Reviewing the entire treatment process, the left heart tumor grew into the left atrium and obstructed flow, thereby presenting with heart failure and high BNP. For this reason, surgery was indicated urgently for the relief of symptoms. But the shortage of this case is the time of PET-CT examination. We could know more about the situation of the patient if the PET-CT was completed before surgery. Since PCT-CT is very expensive, self-funded, and not reimbursed by medical insurance, not everyone can afford it.

Multimodal ultrasound technologies, including two-dimensional echocardiography, color Doppler, and Contrast-enhanced echocardiography, were used to diagnose myxofibrosarcoma and detect hemodynamic changes in this case.

When considering the therapeutic options for primary cardiac myxofibrosarcoma (MFS), it is evident that beyond early diagnosis, identifying the most effective treatment strategy is crucial. Emerging genomic approaches offer promising tools for stratifying patients based on their chemosensitivity, allowing for a more personalized and targeted treatment plan. The study showed that Comparative genomic analysis between myxofibrosarcoma and undifferentiated pleomorphic sarcoma patient samples has pinpointed distinct genetic expressions, with certain genes being up-regulated in UPS and others in MFS (13). Genomic profiling of 7,494 sarcomas, representing a diverse range of histologies, can potentially alter or refine diagnostic outcomes for over 10% of cases. This approach uncovers targetable genetic aberrations in approximately 31.7% of patients, with specific kinase rearrangements noted in 2.6% and a significant tumor mutational burden in 3.9%, thereby enhancing therapeutic decision-making in sarcoma treatment (14). Although this study did not incorporate genomic analysis, we acknowledge the importance of such methodologies and recommend their consideration in future research to enhance our understanding of tumor biology and chemotherapeutic sensitivity.

This study has several limitations that warrant acknowledgment. Firstly, due to financial constraints, a preoperative PET-CT scan was not performed on the patient, which limited our comprehensive assessment of tumor metastasis. Secondly, while we utilized multimodal ultrasonography for detailed tumor evaluation, the study did not include genomic analysis that could have provided further insights into the tumor's characteristics and response to chemotherapy. Additionally, the small sample size may limit the generalizability of our findings to the broader population of MFS patients. Future studies should aim to include a larger cohort and consider integrating genomic analyses to refine treatment strategies.

## Summary

Early detection and treatment of cardiac myxofibrosarcoma are paramount, given its rapid progression and rarity. The integration of echocardiography with contrast-enhanced ultrasound enhances the diagnostic accuracy of cardiac myxofibrosarcoma, facilitating precise diagnosis and aiding in treatment planning. Given the potential for incomplete tumor resection, vigilant follow-up is imperative, and we advocate for echocardiographic monitoring every 3 months. As of the latest update, the patient is still alive and is undergoing regular check-ups.

## Data Availability

The raw data supporting the conclusions of this article will be made available by the authors, without undue reservation.

## Supplementary material

Supplementary Video 1 Contrast-enhanced ultrasonography of the mass. Scan the QR code to watch the video.

Supplementary Video 2 Contrast-enhanced ultrasonography of the recurrent tumor. Scan the QR code to watch the video.
